# Systemic administration of a novel human umbilical cord mesenchymal stem cells population accelerates the resolution of acute liver injury

**DOI:** 10.1186/1471-230X-12-88

**Published:** 2012-07-12

**Authors:** Patrizia Burra, Diletta Arcidiacono, Debora Bizzaro, Tatiana Chioato, Rosa Di Liddo, Antara Banerjee, Andrea Cappon, Patrizio Bo, Maria Teresa Conconi, Pier Paolo Parnigotto, Silvia Mirandola, Enrico Gringeri, Amedeo Carraro, Umberto Cillo, Francesco Paolo Russo

**Affiliations:** 1 Gastroenterology, Department of Surgical, Oncological and Gastroenterological Sciences, Padova University Hospital, Via Giustiniani 2, Padova, 35128, Italy; 2 Department of Pharmaceutical Sciences, University of Padua, Padua, Italy; 3 Obstetrics and Gynecology Unit, Cittadella Hospital, Padua, Italy; 4 VIMM-Venetian Institute of Molecular Medicine, Padua, Italy

**Keywords:** Mesenchymal stem cells, Umbilical cord, Hepatocyte-like cells, Cell transplantation, Acute liver injury, Regenerative medicine

## Abstract

**Background:**

Hepatocytes and stem cells transplantation may be an alternative to liver transplantation in acute or chronic liver disease. We aimed to evaluate the therapeutic potential of mesenchymal stem cells from human umbilical cord (UCMSCs), a readily available source of mesenchymal stem cells, in the CCl_4_-induced acute liver injury model.

**Methods:**

Mesenchymal stem cells profile was analyzed by flow cytometry. In order to evaluate the capability of our UCMSCs to differentiate in hepatocytes, cells were seeded on three different supports, untreated plastic support, Matrigel^TM^ and human liver acellular matrix. Cells were analyzed by immunocitochemistry for alpha-fetoprotein and albumin expression, qPCR for hepatocyte markers gene expression, Periodic Acid-Schiff staining for glycogen storage, ELISA for albumin detection and colorimetric assay for urea secretion.

To assess the effects of undifferentiated UCMSCs in hepatic regeneration after an acute liver injury, we transplanted them via tail vein in mice injected intraperitoneally with a single dose of CCl_4_. Livers were analyzed by histological evaluation for damage quantification, immunostaining for Kupffer and stellate cells/liver myofibroblasts activation and for UCMSCs homing. Pro- and anti-inflammatory cytokines gene expression was evaluated by qPCR analysis and antioxidant enzyme activity was measured by catalase quantification.

Data were analyzed by Mann–Whitney *U*-test, Kruskal-Wallis test and Cuzick’s test followed by Bonferroni correction for multiple comparisons.

**Results:**

We have standardized the isolation procedure to obtain a cell population with hepatogenic properties prior to *in vivo* transplantation. When subjected to hepatogenic differentiation on untreated plastic support, UCMSCs differentiated in hepatocyte-like cells as demonstrated by their morphology, progressive up-regulation of mature hepatocyte markers, glycogen storage, albumin and urea secretion. However, cells seeded on 3D-supports showed a minor or negligible differentiation capacity.

UCMSCs-transplanted mice showed a more rapid damage resolution, as shown by histological analysis, with a lower inflammation level and an increased catalase activity compared to CCl_4_-treated mice.

**Conclusions:**

Our findings show that UCMSCs can be reliably isolated, have hepatogenic properties and following systemic administration are able to accelerate the resolution of an acute liver injury without any differentiation and manipulation. These features make UCMSCs strong candidates for future application in regenerative medicine for human acute liver disease.

## Background

Transplantation is the gold standard procedure for treating acute and chronic end-stage liver disease [1], but the shortage of available organs makes it mandatory to seek alternative therapeutic strategies. Replacing diseased hepatocytes and stimulating endogenous and exogenous regeneration by stem cells represent the main aims of liver-oriented cell therapy [2,3].

Recent developments in stem cell technology have raised the hopes of identifying new expandable sources of liver cells for use in regenerative medicine [4] and prompted studies on the best support for their growth. Embryonic stem cells can be considered the best model of multipotency, but their use is limited due to legal issues, in Italy at least (L. n. 40/2004), as well as safety and ethical concerns [5]. Adult stem cells have consequently been widely explored in recent years as a more acceptable source of cells, including the mesenchymal stem cells (MSCs), a population of multipotent progenitors capable of differentiating towards adipogenic, osteogenic [6], and hepatogenic lineages [7,8] with a low immunogenicity [9]. Therefore this cell population is considered to be a promising candidate for novel cell-based therapeutic strategies [10].

Bone marrow is considered the main source of MSCs [11], but their number decreases significantly with age [12,13] and this has led to the evaluation of alternative sources such as adipose tissue [14] and embryo-derived tissues, e.g. placenta [15], amniotic fluid [16], umbilical cord blood (UCB) [17] and umbilical cord (UC) [18].

UC cells obtained from the sub-endothelial layer of the umbilical vein can differentiate *in vitro* into adipocytes and osteoblasts [19,20], and - when isolated from umbilical cord jelly - they can also differentiate *in vitro* and *in vivo* into a myogenic lineage, as previously reported by our group [21], confirming the presence of plasticity in this population of foetal-derived tissues.

MSC transplantation has been explored as a new clinical approach to repair injured tissue. Following systemic administration MSCs are recruited in the area of ischemia or injury, as was demonstrated in lung [22], heart [23] and kidney [24]. So far, the possibility of using human UCMSCs to repair acute liver damage has not been evaluated.

Therefore, the main aim of this study was to evaluate the therapeutic potential of adult mesenchymal stem cells from human umbilical cord (UCMSCs) in a murine model of acute liver injury using carbon tetrachloride (CCl_4_), a potent hepatotoxic chemical.

More than one protocol has been proposed to isolate these cells without reaching a scientific agreement. It is clearly fundamental to standardize the isolation procedure to obtain an adequate cell population with hepatogenic properties prior to performing successful *in vivo* transplantation. To investigate these hepatogenic capacities we have induced UCMSCs differentiation towards hepatic lineages *in vitro*. Since hepatocytes are known to lose their specific functions rapidly when cultured on a conventional support [25] we sought the best cell support for hepatogenic differentiation.

Stem cell differentiation can be stimulated by growth factors and extracellular matrix (ECM) components are used as a cell culture support. Using an homologous acellular matrix derived from surgical specimens represents an interesting tissue engineering approach since the matrix is biocompatible, contains adhesion molecules and growth factors, and it is obtained from a healthy organ [26,27] and not from hepatoma cell lines, e.g. Matrigel™ [28].

Therefore, we induced UCMSCs hepatic differentiation seeded them on Matrigel^TM^, human liver acellular matrix and on classic petri dishes.

## Results

### UCMSCs **isolation and c**haracterization

**Figure 1:**
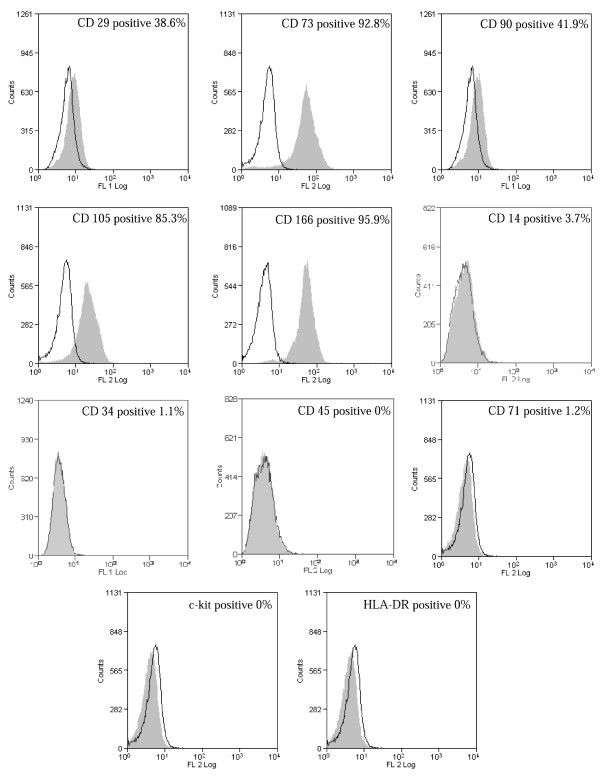
**UCMSCs characterization.** Flow cytometry analysis of UCMSCs showed a mesenchymal phenotype. Cells were positive for typical mesenchymal markers (CD29, CD73, CD90, CD105 and CD166) while hematopoietic markers (CD14, CD34, CD45, CD71 and c-kit) were weakly or not expressed. HLA-DR was not expressed at all

In order to characterize UCMSCs we processed 135 samples of human UC and 98% of them gave rise to cell colonies with fibroblastoid morphology, visible in the culture after 2–3 weeks. Flow cytometry analysis showed a very significant expression of typical mesenchymal cell markers such as CD166, CD105, CD90, CD73 and CD29, while hematopoietic markers (CD14, CD34, CD45, CD71 and c-kit) were weakly or not expressed. HLA-DR was not expressed at all (Figure 1). These results were reproducible when the Wharton jelly fragments were seeded in presence of high glucose DMEM and 20% FBS specific for human MSCs. Moreover, UCMSCs were able to differentiate toward adipogenic and osteogenic lineages (as previously reported by our group [21]). All these results demonstrated that the UCMSCs isolated were a novel mesenchymal stem cell population.

Furthermore, RT-PCR evaluation on UCMSCs obtained from newborn males (representing 53% of our samples) showed the SRY gene expression, confirming the foetal origin of the isolated cells, as previously reported by our group [21].

### *In vitro* UCMSCs hepatogenic differentiation on untreated plastic support

**Figure 2:**
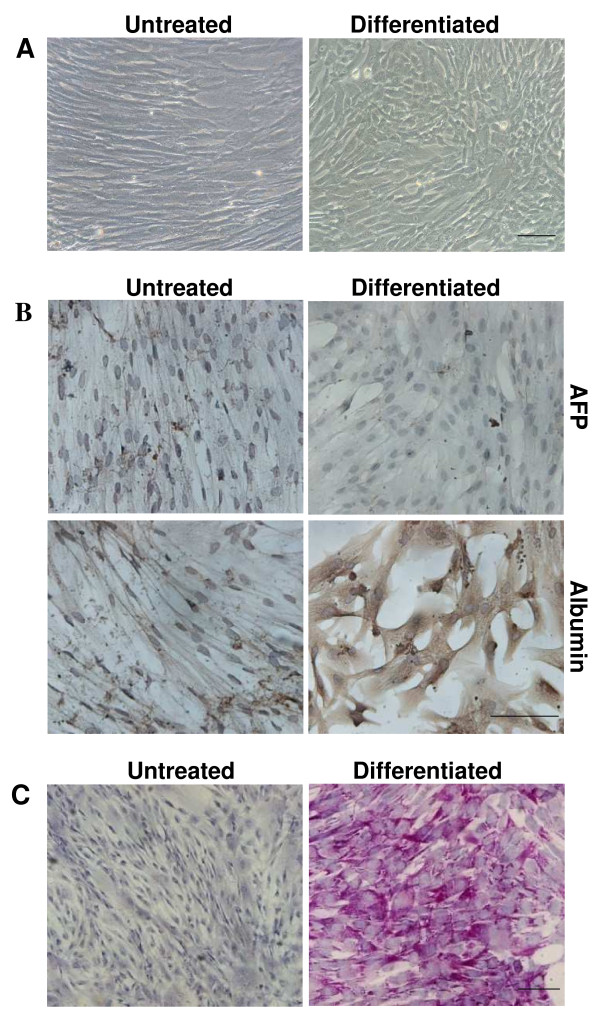
**Characterization of UCMSCs cultured with hepatogenic medium on untreated plastic support.** (**A**) Undifferentiated and differentiated cell morphology. After 14 days of culture in differentiating medium we observed changes in cell morphology: cell spreading was reduced and the UCMSCs acquired a polygonal shape with a granular cytoplasm (**B**) Immunocytochemical analyses for α-fetoprotein (AFP) and albumin in UCMSCs cultured on untreated plastic support. The UCMSCs were cultured on petri dishes with proliferative (untreated) or differentiating medium. AFP protein expression was undetectable in both untreated and differentiated cells. Albumin protein was expressed by differentiated cells after 14 days but not by untreated cells. (**C**) Periodic acid-Schiff (PAS) staining for glycogen storage. UCMSCs cultured with differentiating medium on petri dishes displayed a positive reaction to PAS staining, revealing glycogen storage in their cytoplasm. Untreated cells were negative for PAS staining. Scale bar: 100 μm

In order to evaluate the capability of our UCMSCs to differentiate in hepatocytes, cells were seeded on untreated plastic support up to day 28. After 14 days of culture in differentiating medium we observed changes in cell morphology: cell spreading was reduced and the UCMSCs acquired a polygonal shape with a granular cytoplasm (Figure 2A).

The expression of specific hepatic markers such as the immature hepatoblast marker alpha-fetoprotein (AFP) and the mature hepatocyte markers albumin, microsomal trialcylglycerol transfer protein (MTP) and tryptophan 2–3 dioxygenase (TDO) mRNAs was assessed by qPCR. Unexpectedly, UCMSCs constitutively expressed the messengers coding for hepatic markers generally attributed to mature hepatocytes, suggesting their hepatogenic potentiality.

In control UCMSCs hepatic markers expression remained stable at all time points considered (day 7, 14, 21 and 28) as confirmed by the Cuzick’s trend test. Otherwise, mRNA expression of these markers was modulated in the presence of differentiating factors (Table 1).

**Table 1:** qPCR analysis for hepatic markers in differentiated UCMSCs

	**7 days**	*p	**14 days**	*p	**21 days**	*p	**28 days**	*p	^¥^**p**
**Petri dishes**									
AFP	0.481	0.0402	0.573	0.0377	0.358		0.124	0.0003	0.0378
	(0.472; 0.482)		(0.566; 0.575)		(0.357; 0.364)		(0.122; 0.124)		(z = −2.08)
Alb	2.652		4.298		5.991		7.880		0.0219
	(2.630; 2.683)		(4.276; 4.438)		(5.966; 6.261)		(7.718; 8.144)		(z = 2.30)
MTP	1.012	NS	1.924		3.606		4.805		0.0021
	(1.007; 1.017)		(1.910; 1.929)		(3.521; 3.612)		(4.741; 4.821)		(z = 3.08)
TDO	7.464		10.483		16.111		19.575		0.0023
	(7.380; 7.576)		(10.301; 10.601)		(16.040; 16.295)		(19.497; 19.802)		(z = 3.12)
**Matrigel**^**TM**^									
AFP	0.719	0.0450	0.592	NS	0.638	0.0202	0.587		NS
	(0.715; 0.719)		(0.591; 0.596)		(0.635; 0.639)		(0.583; 0.589)		
Alb	0.704		1.437	NS	1.926	NS	2.486		0.0065
	(0.701; 0.704)		(1.419; 1.445)		(1.895; 1.945)		(2.446; 2.520)		(z = 2.72)
MTP	0.789	NS	0.814	NS	2.452		2.274		0.0122
	(0.781; 0.790)		(0.813; 0.820)		(2.432; 2.461)		(2.258; 2.278)		(z = 2.51)
TDO	1.250	NS	1.210	NS	1.269	NS	1.490	NS	NS
	(1.170; 1.317)		(1.140; 1.374)		(1.258; 1.428)		(1.443; 1.594)		
**hLAM**									
AFP	0.992	NS	0.823	NS	0.778	NS	0.871	NS	NS
	(0.982; 0.994)		(0.819; 0.829)		(0.777; 0.788)		(0.837; 0.876)		
Alb	1.580	0.0353	0.860	NS	0.834	NS	0.788	NS	NS
	(1.570; 1.595)		(0.859; 0.867)		(0.822; 0.847)		(0.786; 0.793)		
MTP	nd		nd		nd		nd		
TDO	nd		nd		nd		nd		

*p Mann–Whitney *U*-test ^¥^p Cuzick’s trend test.

Values represent median (Q1; Q3) of 5 independent experiments. Data were analyzed using the second-derivative algorithm. For each sample, the quantity of specific hepatic marker mRNA was expressed as n-fold the normalized amount of mRNA from undifferentiated control cells.

The Mann–Whitney *U*-test was used for the statistical analysis of differences between undifferentiated control cells and differentiated cells at each time points (*p). Multiple comparisons to evaluate the changes of a single mRNA expression during the time were performed with the Cuzick’s trend test (^¥^p). nd: not detected; NS: not significant.

In fact, the expression of AFP in differentiating conditions was significantly lower than in control cells at each time point (*p values shown in Table 1). This down-regulation was significant during the time course (^¥^p = 0.0378 z = −2.08).

In contrast, the expression of albumin mRNA was significantly increased at each time point (*p values reported in Table 1) and this up-regulation was time dependent (^¥^p = 0.0219 z = 2.30).

Levels of MTP mRNA were significantly up-regulated only from the 14^th^ day onwards and this increase was time-dependent, as confirmed by Cuzick’s trend test (^¥^p = 0.0021 z = 3.08). TDO (an enzyme belonging to the family of oxidoreductases) mRNA expression was significantly up-regulated at each time point in a time-dependent manner (^¥^p = 0.0023 z = 3.12) (Table 1).

Immunocytochemistry performed using specific anti-human albumin antibodies clearly demonstrated the presence of albumin already after 14 days of differentiation (while the protein was never detected in control cells). No AFP expression was detectable in either the differentiated or the control cells (Figure 2B).

Differentiation efficiency was also assessed in functional terms. ELISA confirmed that differentiated cells were able to secrete albumin in a time-dependent manner (^¥^p = 0.0002; z = 3.75), as shown in Table 2.

**Table 2:** Albumin secretion in differentiated UCMSCs

	**7 days**	**14 days**	**21 days**	**28 days**	^**¥**^**p**
Petri dishes	2.135	6.565	7.850	10.668	0.0002
	(2.010; 2.345)	(6.512; 6.642)	(7.725; 7.925)	(10.525; 10.760)	(z = 3.75)
Matrigel	1.690	6.990	6.580	10.230	0.0010
	(1.627; 1.772)	(6.422; 7.607)	(6.400; 6.890)	(10.082; 10.407)	(z = 3.28)
hLAM	1.475	1.740	2.825	2.985	0.0006
	(1.382; 1.562)	(1.677; 1.822)	(2.677; 3.117)	(2.852; 3.232)	(z = 3.45)

^¥^p Cuzick’s trend test.

Values represent median (Q1; Q3) (ng/ml) of 5 independent experiments. Time trends were evaluated using the Cuzick’s trend test.

Periodic acid-Schiff staining also showed that the UCMSCs subjected to the hepatogenic differentiation protocol were able to store glycogen after 14 days of culture, while the undifferentiated UCMSCs did not (Figure 2C).

Urea measured after 28 days of culture in hepatogenic medium showed that cells were able to metabolize ammonia [1.222 (1.134; 1.304) mM/10^4^cells/day]. Undifferentiated cells produced lower levels of urea [0.284 (0.273; 0.328) mM/10^4^cells/day]. Mann Whitney-*U* test showed that this difference was statistically significant (*p = 4.246x10^-5^).

Finally, flow cytometry analysis of the differentiated cells demonstrated a decreased expression of the typical MSCs markers, such as CD73, CD90 and CD105, except CD29 and CD166, acquiring a phenotype which was more compatible with a mature cell (Table 3).

**Table 3:** Characterization of UCMSCs after 28 days of culture

	**Undifferentiated UCMSCs**	**Differentiated UCMSCs**
**MSC Markers**		
CD29	53.61%	79.4%
CD73	97.3%	78.8%
CD90	43.15%	32.8%
CD105	72.6%	13.7%
CD166	61.5%	96.5%
**HSC Markers**		
CD14	0%	0%
CD34	0.7%	7.3%
CD45	1.2%	1.3%
CD71	10.4%	4.5%
c-kit	0%	5.8%
HLA-DR	0%	0%

Flow cytometry analysis of differentiated UCMSCs on untreated plastic support after 28 days showed a lower expression of the typical MSC markers, such as CD73, CD90 and CD105. CD166 was spontaneously lost in undifferentiated cells whereas its expression was maintained in differentiated UCMSCs. CD29 expression in differentiated cells increased to reach a level comparable with human hepatocytes [7]. Hematopoietic markers remained stable for up to 28 days both in differentiated and untreated cells.

### *In vitro* UCMSC hepatogenic differentiation on Matrigel™

**Figure 3:**
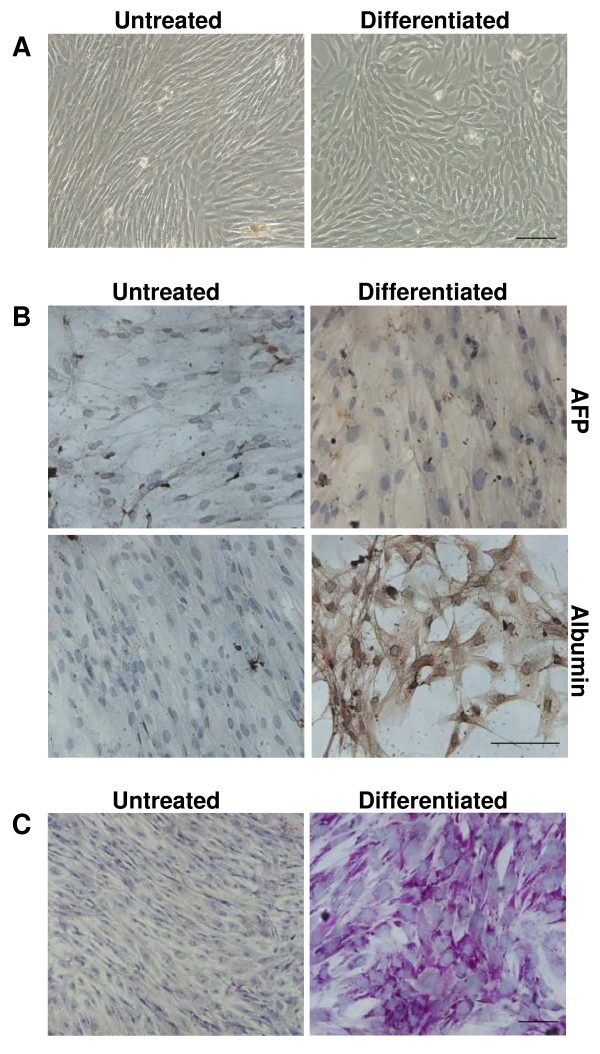
**Characterization of UCMSCs cultured with hepatogenic medium on Matrigel™.** (**A**) Undifferentiated and differentiated cell morphology. Optical analyses showed morphological changes after 14 days in hepatogenic medium. (**B**) Immunocytochemical analyses for α-fetoprotein (AFP) and albumin of UCMSCs cultured on Matrigel™ with proliferative or differentiating medium. AFP was never detected in either proliferative (untreated) or differentiating medium. Albumin protein was expressed only by differentiated cells after 14 days. (**C**) PAS staining for glycogen storage. UCMSCs cultured with differentiating medium on Matrigel™ displayed a positive reaction to PAS staining, revealing glycogen storage in their cytoplasm. Untreated cells were negative for PAS staining. Scale bar: 100 μm

In order to test whether a biological 3D support could influence the UCMSCs differentiation toward hepatocytes, cells were seeded on Matrigel™. Optical analyses showed morphological changes after 14 days in hepatogenic medium, as seen in the cells differentiated on untreated plastic support (Figure 3A).

qPCR showed that expression of hepatic markers AFP, albumin and MTP, with the exception of TDO messenger, was modulated during the differentiation process at each time point.

AFP mRNA expression in UCMSCs cultured in differentiating medium was significantly lower than in the corresponding control cells, but it remained stable up to 28 days (Table 1).

Although initially down-regulated, albumin mRNA expression showed a rising trend, but this up-regulation was only significant at 28^th^ day of differentiation. Cuzick’s trend test showed that up-regulation were time-dependent (^¥^p = 0.0065 z = 2.72).

MTP mRNA expression was significantly up-regulated from the 21^st^ day onwards in comparison with the control values and this increase was time-dependent (^¥^p = 0.0122 z = 2.51). TDO mRNA expression remained stable compared to control cells and also during the differentiating process (Table 1).

As previously obtained with the untreated plastic support, immunocytochemistry analyses showed that albumin protein was only expressed by the differentiated cells after 14 days and no AFP expression was detectable in the differentiated or in control cells (Figure 3B).

Functional tests demonstrated that albumin was secreted in the supernatants of differentiated UCMSCs cultures in a time-dependent manner (^¥^p = 0.0010; z = 3.29), reaching levels comparable with those of the differentiated cells seeded on untreated plastic support (Table 2).

No glycogen storage was observed in the undifferentiated cells, whereas the UCMSCs cultured with differentiating medium were positive to PAS staining already after 14 days (Figure 3C).

Urea measured after 28 days of culture in hepatogenic medium showed that cells were able to metabolize ammonia in a comparable fashion with those measured on cells differentiated on untreated plastic support [1.197 (1.161; 1.240) mM/10^4^cells/day]. Undifferentiated cells produced lower levels of urea [0.292 (0.257; 0.309) mM/10^4^cells/day]. Mann Whitney-*U* test showed that this difference was statistically significant (*p = 2.538x10^-5^).

### *In vitro* UCMSCs hepatogenic differentiation on hLAM

In order to verify the differentiating capability of UCMSCs *in vitro* on a more adapt biological support, we seeded the cells on human liver acellular matrix.

Preliminary histochemical and scansion electronic microscopy (SEM) analyses showed that the matrix sections obtained from human liver were completely decellularized after a single treatment cycle. Masson’s trichrome staining also confirms that the matrix retained its fibrillary components after the treatment (data not shown).

qPCR analysis showed that the AFP and albumin mRNA expression in UCMSCs seeded on hLAM either in proliferative and differentiating medium remained stable during the time. MTP and TDO mRNA were never expressed in either proliferative or differentiating conditions (Table 1).

Albumin detection in the supernatant of UCMSCs cultured with the differentiating protocol showed a significant increase during the time considered (^¥^p = 0.0006 z = 3.45), but the amount of protein secreted was always significantly lower than with the other supports (Tables 2–4). Both in proliferative and differentiative conditions cells produced low and comparable levels of urea [0.244 (0.202; 0.287) mM/10^4^cells/day and 0.270 (0.230; 0.299) mM/10^4^cells/day, respectively. *p = NS].

**Table 4:** mRNA expression and albumin secretion at each time-point

**A) Kruskal-Wallis test**					
		7 days	14 days	21 days	28 days
AFP		0.0493	NS	0.0491	NS
Alb		0.0332	0.0273	0.0391	0.0394
MTP		nd	nd	nd	nd
TDO		nd	nd	nd	nd
ALB secretion		0.0125	0.0231	0.0248	0.0210
B) Kruskal Wallis test followed by Bonferroni correction					
		7 days	14 days	21 days	28 days
Petri dishes vs. hLAM	AFP	0.0153	NS	0.0138	0.0040
	Alb	NS	0.0014	0.0034	0.0010
	MTP	nd	nd	nd	nd
	TDO	nd	nd	nd	nd
	Alb secr	0.0009	p < 0.0001	p < 0.0001	p < 0.0001
Matrigel^TM^ vs. hLAM	AFP	NS	NS	NS	NS
	Alb	0.0039	NS	NS	NS
	MTP	nd	nd	nd	nd
	TDO	nd	nd	nd	nd
	Alb secr	NS	p < 0.0001	p < 0.0001	p < 0.0001
Petri dishes vs. Matrigel^TM^	AFP	NS	NS	NS	0.0118
	Alb	0.0003	0.0082	NS	NS
	MTP	NS	0.0026	NS	0.0127
	TDO	p < 0.0001	p < 0.0001	p < 0.0001	p < 0.0001
	Alb secr	0.0088	NS	NS	NS

(**A**) The Kruskal-Wallis test was used to evaluate the differences between the three supports. (**B**) After Bonferroni correction p < 0.016 was assumed as significant. nd: not detected; NS: not significant.Comparison between the supports.

### Comparison between supports: untreated plastic is most suitable

To assess the differences between the three supports considered in our study we performed a multiple comparison of the expression levels of each marker analyzed and the albumin secretion at each time point. This analysis clearly suggested a difference between the supports (Table 4A). The Kruskal-Wallis followed by Bonferroni correction demonstrated that hLAM was the most unsuitable support for the purpose of UCMSCs differentiation. In fact, the pairwise comparison showed that the AFP mRNA down-regulation and albumin mRNA up-regulation were more marked in petri dishes. Furthermore, the albumin secretion was unequivocally stronger when the cells were seeded on petri dishes or on Matrigel^TM^ rather than seeded on hLAM. In addition, the absence of any MTP and TDO mRNA, suggest that the UCMSCs cultured on hLAM failed to reach the hepatocyte-like phenotype (Table 4).

Pairwise comparison between petri dishes and Matrigel™ brings out that the AFP mRNA down-regulation was more pronounced on petri dishes and reaching significant levels at 28^th^ day of differentiation (^§^p = 0.0118).

Finally, MTP mRNA up-regulation – which was always time-dependent - seemed to be more marked in the cells seeded on untreated plastic support (^¥^p = 0.0021 vs. ^¥^p = 0.0122). The multiple comparison followed by Bonferroni correction showed that MTP mRNA induction after 14 and 28 days was significantly greater on petri dishes. Interestingly, TDO mRNA up-regulation was verified exclusively when the cells were seeded on untreated plastic support.

### Transplanted UCMSCs accelerate the recovery after CCl_4_-induced liver injury

In order to evaluate the therapeutic potential of the UCMSCs in an acute liver damage we transplanted them into CCl_4_-treated mice. Considering their hepatocyte-like phenotype and their capability to differentiate in hepatocyte-like cells we transplanted undifferentiated UCMSCs.

**Figure 4:**
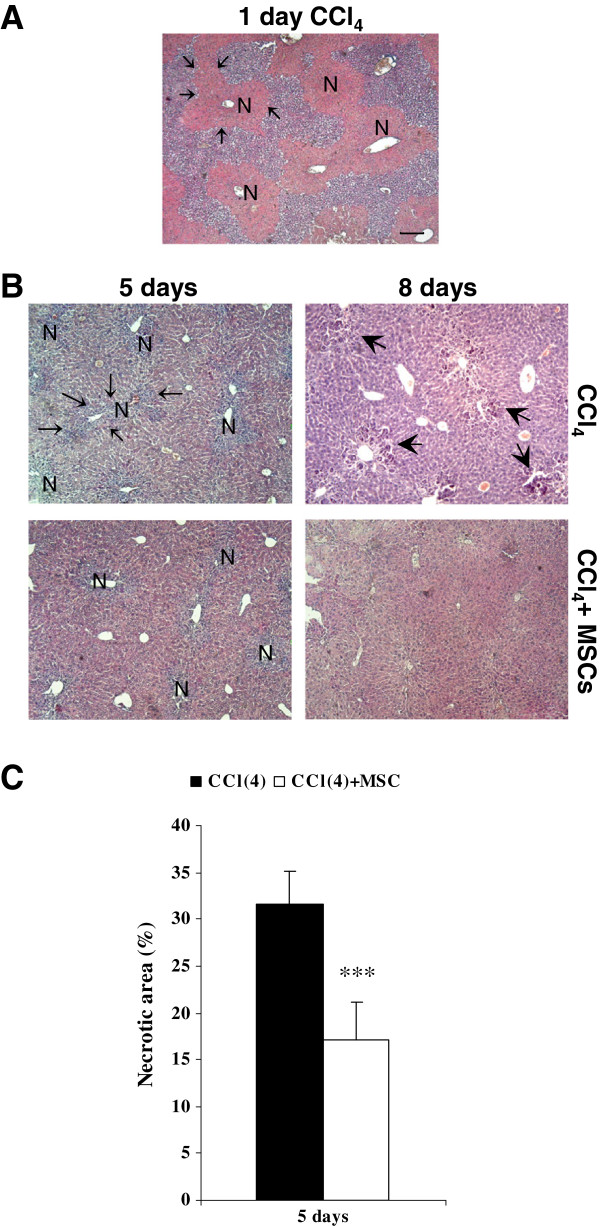
**CCl**_**4**_**-induced liver injury and liver recovery after UCMSCs transplantation.** (**A**) Haematoxylin and eosin stain. After 24 hours from CCl_4_ injection confluent coagulative submassive necrosis affected 43.3% of the liver. The margins of representative necrotic area (N) are indicated by arrows. (**B**) Haematoxylin and eosin stain. After 5 days from CCl_4_ administration the necrotic area (N, indicated by arrows) was still wide and there was a conspicuous inflammatory infiltrate. After 8 days there were not necrotic areas and numerous cellular clusters were evident (head arrows). See Additional file 1: Figure S1 for higher magnification. UCMSCs transplantation in CCl_4_-treated mice reduced the amount of inflammatory cells and necrosis area after 5 days. Necrosis, inflammatory recruited cells and cellular cluster were completely absent after 8 days and histological pattern was becoming similar to control healthy mice parenchyma (see Additional file 2: Figure S2). (**C**) Necrosis quantification at day 5. Scale bar: 100 μm

**Figure 5:**
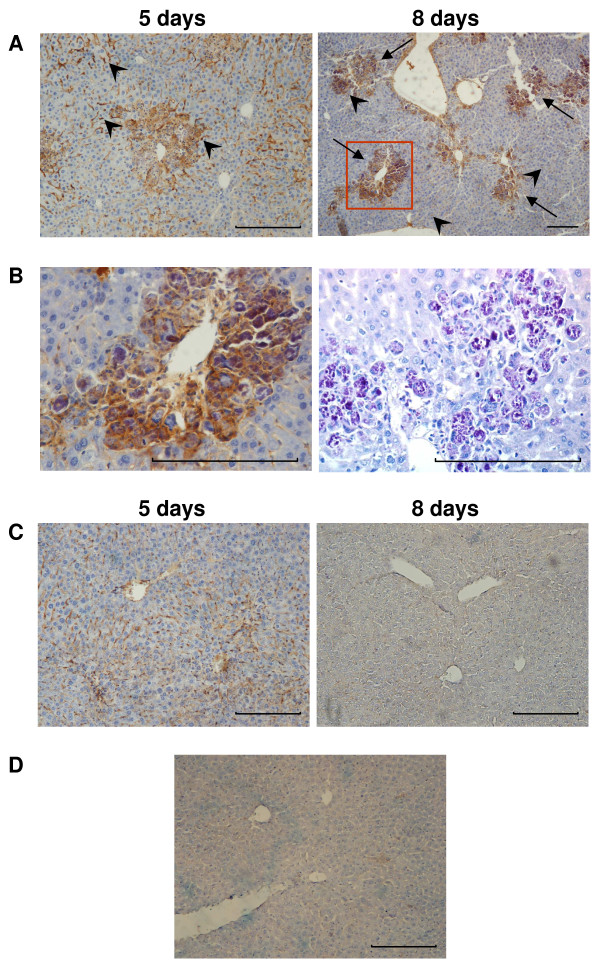
**Identification of CD68 positive cells in liver.** (**A**) CCl_4_-treated mice. CCl_4_ induced Kupffer cells (representative cells indicated by head arrows) activation at day 5. At day 8 were also evident activated mega macrophages (arrows) that were organized to form cellular clusters localized exclusively in necrotic areas. (**B**) High magnification of CD68 positive cellular clusters (red square in A). Mega macrophages were endowed with phagocytic activity as demonstrated by PAS-D stain (right image). (**C**) UCMSCs transplantation in CCl_4_-treated mice. The amount of CD68 positive cells was reduced at day 5 compare to CCl_4_-trated mice. After 8 days there were few CD68 positive cells, as detected in control mice liver. (**D**) Control (PBS) mice liver. Scale bars: 200 μm

CCl_4_ exposure induced confluent coagulative submassive necrosis and disorganized the normal architecture of parenchyma (Figure 4A). After 5 days from CCl_4_ administration, the necrotic area was 31.27% of parenchyma, was visible a conspicuous inflammatory infiltrate (Figure 4B-C and higher magnification in Additional file 1: Figure S1) and a large amount of activated Kupffer cells. At 8 day, the necrotic areas were absent and totally replaced by numerous cellular clusters that we identified as activated mega macrophages and Kupffer cells. Indeed, these cells were CD68 positive and were endowed with phagocytic activity as demonstrated by PAS-D stain (Figure 5A-B).

UCMSCs transplantation in CCl_4_-treated mice reduced the amount of inflammatory cells at day 5 and the necrotic areas were about 17.2% of parenchyma. Necrotic areas, inflammatory recruited cells and activated mega macrophages were completely absent after 8 days (Figures 4 and 5). Therefore histological pattern developed characteristics similar to those of control healthy mice parenchyma (Additional file 2: Figure S2).

**Figure 6:**
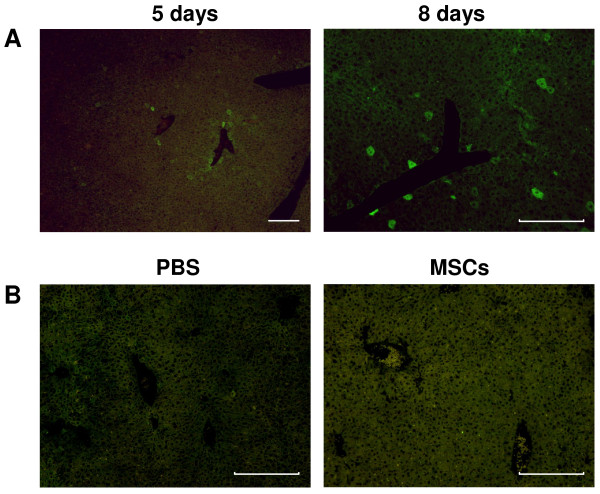
**Identification of human albumin positive cells in transplanted mice liver.** (**A**) Immunofluorescence analysis showed positive cells for human albumin in livers of CCl_4_-treated mice that had undergone UCMSCs transplantation both at day 5 and 8 from the damage. (**B**) Mice livers from control groups did not show any human albumin positive cells. Scale bars: 200 μm

Immunofluorescence analysis showed positive cells for human albumin in livers of CCl_4_-treated mice that had undergone UCMSCs transplantation both at 5 and 8 days from the damage (Figure 6A). No cells were found in transplanted mice without liver damage (Figure 6B).

### Transplanted UCMSCs were able to reduce liver inflammation and to inhibit stellate cell and myofibroblasts activation

**Figure 7:**
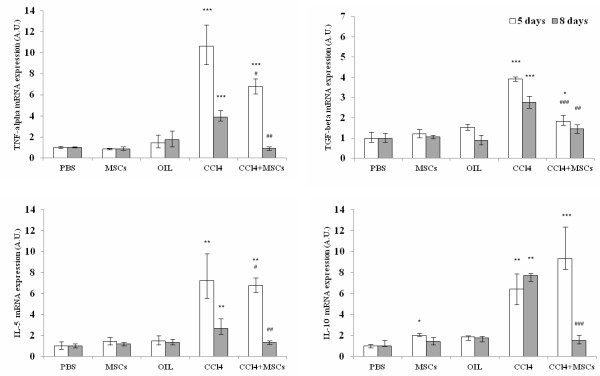
**mRNAs expression of pro-inflammatory and anti-inflammatory cytokines*in vivo.*** CCl_4_ administration induced significant up-regulation of mRNA coding for TNF-alpha, TGF-beta 1, IL-5 and IL-10. Following cell transplantation, at both 5 and 8 days from the injury the pro-inflammatory cytokines mRNA up-regulation was significantly lower compared to mice that received only CCl_4_. IL-10 mRNA up-regulation at 5 days was significantly higher compared to CCl_4_ group and returned to basal level after 8 days from the damage. Values represent median, negative and positive error bars represent Q1 and Q3, respectively. * Mann Whitney *U* test vs. PBS group; # Mann Whitney *U* test vs. CCl_4_ group *p < 0.05; ** or ## p < 0.01; *** or ### p < 0.001

To confirm histological analysis, we evaluated the inflammatory state quantifying mRNAs expression of pro-inflammatory (TNF-alpha, IL-5 and TGF-beta 1) and anti-inflammatory cytokines (IL-10) by qPCR. CCl_4_ administration induced significant up-regulation of mRNA coding for all considered cytokines at both time points. Following cell transplantation, both at 5 and 8 days from the damage, qPCR analysis showed that the pro-inflammatory cytokines mRNA up-regulation was significantly lower compared to mice that received only CCl_4_, whereas IL-10 mRNA up-regulation at 5 days was significantly higher compared to CCl_4_ group and returned to basal level after 8 days from the damage (Figure 7).

**Figure 8:**
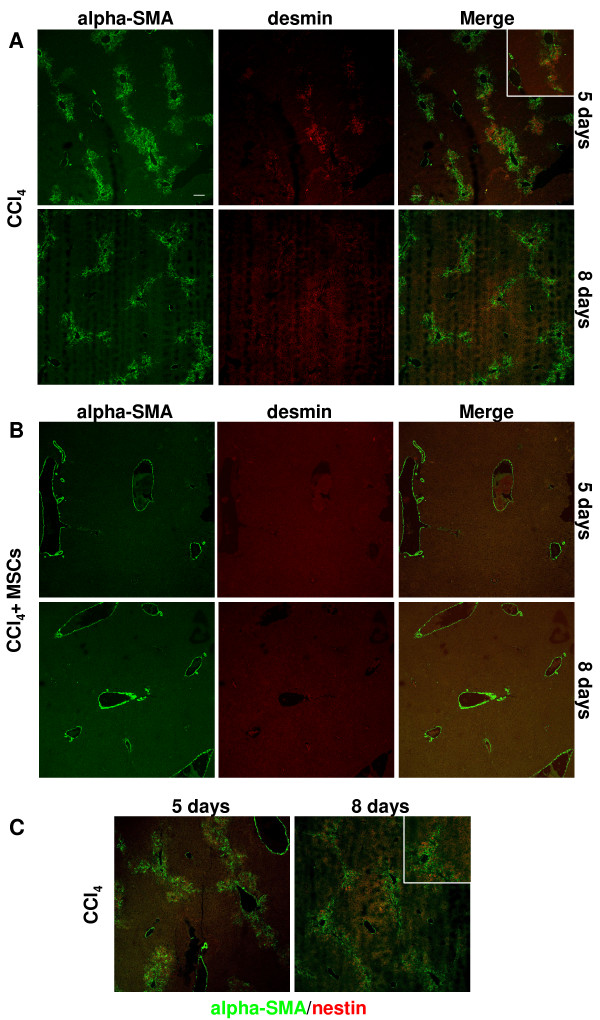
**Stellate cells and myofibroblasts activation in CCl**_**4**_**-treated mice with and without UCMSCs transplantation.** (**A**) Co-staining alpha-SMA/desmin in CCl_4_-treated mice. A conspicuous number of alpha-SMA (smooth muscle actin) positive cells expressed also desmin identifying them as stellate cells. Alpha-SMA single positive cells were myofibroblasts. At day 8 both cellular types were significantly decreased. (**B**) Co-staining alpha-SMA/desmin in CCl_4_-treated mice that had undergone UCMSCs transplantation. Both at day 5 and 8 no positive cells for alpha-SMA except vascular smooth muscle cells were detected. Few desmin positive cells were visible. (**C**) Co-staining alpha-SMA/nestin in CCl_4_-treated mice. Localization and quantity of double positive cells were comparable to alpha-SMA/desmin positive cells confirming stellate cells identity. The images inside the white square represent a zoomed detail (see higher magnification on Additional file 3: Figure S3). Scale bar: 100 μm

TGF-beta 1 modulation was correlated with activation/deactivation of stellate cells and myofibroblasts. Immunofluorescence showed that in CCl_4_-treated mice there was a conspicuous number of alpha-SMA (smooth muscle actin) positive cells that significantly decreased during the time [120.54 (117.90-122.13) and 102.16 (94.39-105.11) cells/field at day 5 and 8 respectively; *p = 0.048]. To evaluate the percentage of stellate cells within totality alpha-SMA positive cells, co-straining with desmin was performed. Desmin, an intermediate filament typical of contractile cells, has been widely used as a "gold standard" for identifying stellate cells in rodent liver [29]. At day 5, 80.55% of alpha-SMA positive cells co-expressed desmin, decreasing to 48.03% at day 8. To further confirm the identity of stellate cells, co-staining alpha-SMA/nestin was performed. It is known that nestin, a class VI intermediate filament protein, is induced during stellate cells activation in rodent liver [30]. Localization and quantity of alpha-SMA/nestin double positive cells (77.27% and 53.34% at day 5 and 8, respectively) were comparable to alpha-SMA/desmin positive cells (Figure 8). Higher magnification of double positive cells were showed in Additional file 3: Figure S3.

On the contrary, in UCMSCs transplanted mice there were not positive cells for alpha-SMA except vascular smooth muscle cells and few desmin and nestin positive cells were visible (Figure 8).

### UCMSCs influenced liver antioxidant enzyme activity

**Figure 9:**
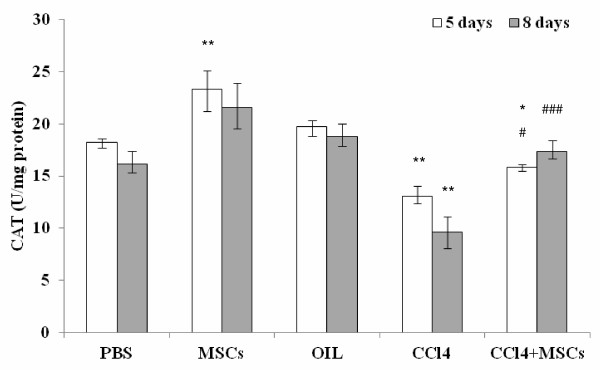
**Liver catalase activity.** A significant reduction in the activity of catalase was observed in the liver tissue of the CCl_4_-intoxicated experimental animals. Transplantation of UCMSCs both alone and after CCl_4_ administration, increased catalase activity compared to respective control groups. The injection of oil alone did not influence catalase activity. Values represent median, negative and positive error bars represent Q1 and Q3, respectively. * Mann Whitney *U* test vs. PBS group; # Mann Whitney *U* test vs. CCl_4_ group * or # p < 0.05; **p < 0.01; ### p < 0.001

Antioxidant enzymes are considered to be the first line of cellular defence that prevents cellular components from oxidative damage. Among them, superoxide dismutase (SOD) and catalase mutually function as important enzymes in the elimination of ROS (reactive oxygen species). A significant reduction in the activity of catalase was observed in the liver tissue of the CCl_4_-intoxicated experimental animals. Transplantation of UCMSCs alone induced an increase of catalase activity (significant at day 5) compared to PBS (Phosphate Buffered Saline) group. Transplantation of UCMSCs after CCl_4_ administration increased catalase amount (reaching control values at day 8) compared to CCl_4_ alone group (Figure 9).

## Discussion

Studies on stem cells and on their potential sources have been intensified in recent years, given the promise of their clinical application, especially in regenerative medicine [31].

Ethical problems regarding the use of human embryos [5] and the neoplastic risks after their *in vivo* use [32] have led to adult stem cells being considered a more acceptable source. Bone marrow is a good source of adult stem cells but the decrease in number of stem cells available with the donor age and invasive procedure required to obtain the cells are the major problems for their utilization [12,13]. Research has consequently turned towards finding alternative sources of MSCs, such as adipose tissue [14] and foetal-derived tissues. Placenta, amniotic fluid and umbilical cord indeed seem to contain undifferentiated cells, due to their embryonal origin [15-18].

In this study we characterized a novel MSCs population obtained from human UC (UCMSCs) and we have induced their differentiation towards hepatic lineages *in vitro* seeking the best cell support for this purpose*.*

Phenotypic analysis showed a profile compatible with MSCs and the simultaneous high expression of CD166, CD105 and CD73 demonstrated that our cells were a novel MSCs population.

We found that these UCMSCs constitutively express mRNA coding for specific hepatic markers such as AFP, albumin and TDO. Then, for the first time, we unexpectedly demonstrated that undifferentiated UCMSCs constitutively expressed mRNA coding for MTP, a transfer protein localized in the endoplasmic reticulum of mature hepatocytes and enterocytes; this protein catalyses the transport of triglycerides, cholesteryl esters and phospholipids [33].

We believe that these findings reinforce the conviction that UCMSCs have a strong potential for differentiating into hepatic-lineage cells *in vitro*.

In fact, when UCMSCs were stimulated to differentiate towards a hepatic lineage, reached a hepatocyte-like phenotype, amazingly, when seeded on a simple untreated plastic support.

Further confirmation comes from flow cytometric analysis on differentiated cells that demonstrated a conspicuous drop in almost all the typical MSCs markers, such as CD73, CD90, and CD105. The only exception was CD166 expression that was spontaneously lost in UCMSCs cultured in proliferative medium while was maintained when the cells were submitted to differentiation protocol. Interestingly, CD29, another typical MSC marker, was expressed higher in differentiated UCMSCs (79,4%) reaching a level comparable with human hepatocytes [7].

The morphological features, loss of MSC phenotype, gene expression changes, immunocytochemical staining, albumin secretion, urea production and glycogen storage, all suggested that these cells can grow and differentiate into functional hepatocyte-like cells without any biological support, whereas cells seeded on 3D-supports showed a minor (Matrigel^TM^) or negligible (hLAM) differentiation capacity. Indeed, the data that unquestionably confirm our assertions was on TDO mRNA modulation that was found exclusively when the cells were differentiated on petri dishes. Our data would indicate that hLAM is not a suitable support for cell growth and hepatic differentiation considering that the cells lost mRNA coding for MTP and TDO after 7 days of culture and the supplementation with hepatogenic differentiating factors did not influence the basal AFP and albumin mRNA levels during 28 days of stimulation. These findings suggest that, in our experimental conditions, the coating afforded no advantage over an untreated plastic support for the purpose of UCMSCs hepatic differentiation; on the contrary, the simplest support seems to be the most suitable for this aim.

Taken together these results demonstrated that this novel population is an ideal candidate for liver disease treatment by cell therapy.

Mesenchymal stem cell transplantation has been explored as a new clinical approach to repair injured tissue. A growing number of studies have highlighted two important aspects of MSC therapy: MSCs can modulate T-cell mediated immunological responses [9,34] and following systemic administration these cells home to sites of ischemia or injury, as was demonstrated in lung [22], heart [23], kidney [24] and liver [35]. Several different MSC sources have been evaluated for cell therapy in chronic and acute liver diseases, such as bone marrow [36], amniotic fluid [37] and human umbilical cord blood [38,39]. On the contrary, UCMSCs have been exclusively considered for therapy in chronic liver diseases, such as fibrosis and cirrhosis, and so far liver UCMSCs transplantation *in situ* has been the unique administration route [40,41].

It has been documented that multipotent MSCs synthesize a wide variety of growth factors and cytokines, exerting a paracrine effect on local cellular dynamics [42]. Such trophic effects could be irrespective of direct differentiation of transplanted cells into lineages of the respective tissues as demonstrated in an ischemic acute renal failure model [43]. The hepatogenic potential and immunomodulatory activity of UCMSCs were further investigated in this study employing systemic transplantation in a murine model of acute liver injury induced by a single administration of carbon tetrachloride, able to induce severe hepatic damage by generation of oxidative stress and activation of immune cells [44]. In fact, so far the possibility of using human UCMSCs to repair acute liver damage has not been evaluated and they have been transplanted via systemic administration by our group for the first time. MSCs were found to be very resistant to ROS and induced a faster reduction of oxidative stress in recipient mice [35]. For these reasons, we decided to transplant undifferentiated UCMSCs rather than pre-differentiated cells.

We transplanted UCMSCs after 24 hours from the damage, when 40% of hepatic parenchyma was necrotic. Cells were recruited in the injured tissue and then they were able to engraft the liver (cells did not reach the liver when transplanted in healthy mice) and to regulate the inflammatory process. In fact in transplanted mice as soon as 5 days after the CCl_4_ injection, the inflammatory process was clearly attenuated, showing a moderated infiltrate, a lower CD68 positivity, a lower pro-inflammatory cytokines expression (primarily TNF-alpha and TGF-beta 1) and a higher level of IL-10 gene expression compared to the CCl_4_ alone group.

At day 8 histological analysis of liver of CCl_4_ treated mice showed the presence of cellular clusters that we identified as mega macrophages and activated Kupffer cells. In transplanted mice it was possible to identify exclusively Kupffer cells only at day 5, whereas after 8 days this activation phase was completely terminated. These findings suggest that transplanted UCMSCs have anti-inflammatory properties or are able to accelerate the kinetic of inflammatory process, leading to liver recovery in a shorter time.

During the pro-inflammatory process induced by CCl_4_, jointly with Kupffer cells, stellate cells play a pivotal role. Immunofluorescence analysis showed that in CCl_4_-treated mice there was a conspicuous number of alpha-SMA positive cells. The most of these cells were identified as activated stellate cells since they co-expressed desmin and nestin. Alpha-SMA single positive cells were another subpopulation of liver myofibroblasts. Following cells transplantation there was no activation of stellate cells, therefore, the anti-inflammatory activity of UCMSCs was directed not only towards inflammatory cells, including Kupffer cells but also against stellate cells and myofibroblasts. TGF-beta 1 down-regulation in transplanted mice confirmed our hypothesis.

The liver has several antioxidant enzymatic systems such as superoxide dismutase, catalase and glutathione peroxidase that play a fundamental role during physiological and pathological ROS mediated oxidative stress. We evaluated whether UCMSCs were able to ameliorate hepatic damage also influencing the antioxidant systems, measured by catalase activity within the liver. In CCl_4_ treated mice livers the activity of catalase was significantly reduced compared with control groups. The enzyme was probably degraded or saturated to block CCl_4_-induced massive free radical production. In presence of mesenchymal stem cells catalase activity was higher after 8 days compared to CCl_4_ treated mice. These finding suggested that UCMSCs reduced catalase consumption, confirming that oxidative damage in transplanted mice was nearly resolved.

Our experiments also showed that catalase activity measured in the liver of UCMSCs transplanted mice without any CCl_4_ induced damage (MSCs group) was higher compared to PBS group. Therefore we speculate that UCMSCs contribute to scavenging activity against radicals by stimulating the activity of catalase, one of the biological defence system of the liver.

*In vivo* experiments showed that unquestionably UCMSCs were able to induce total liver recovery acting as an adjuvant or modulating the physiologic defence systems. There is not a direct relationship between the number of cells that are found in the liver and the amazing results in transplanted mice. It was demonstrated that stem cells act through a dual mechanism: cell-to cell contact and modulation mediated by soluble factors produced by cells themselves. Our hypothesis is that cells have immunomodulatory activity both at local level and before homing to damaged tissue. It is possible that cells are able to act also during their permanence in the bloodstream and completing their action upon reaching the liver. Increased catalase activity measured in MSCs group demonstrated that cells were able to influence hepatic antioxidant environment without liver engraftment.

## Conclusions

Our results look promising for future application of UCMSCs in regenerative medicine and clinical practice. The chances of using such cells in this field are increased by the simplicity and reproducibility with which a significant number of cells can be obtained, by the homogeneous features between different cellular preparations that need not to undergo any sorting, by the absence of immunogenicity (because UCMSCs are HLA-DR-negative) and, most importantly, by the fact that they carry no legal or ethical implications. Cell transplantation is a practical procedure compared with organ transplantation. It can be performed with much less risk to the patient and much reduced cost for the healthcare system. Furthermore, given the little invasiveness of systemic administration, this method could be also applied to patients who are severely ill and would not be able to tolerate organ transplantation. We are aware that the conveyance of our results into clinical practice would need to be considered with caution because more information is needed on these cells’ behavior *in vivo* before any clinical applications can be hypothesized, but the potential shown by our UCMSCs is undeniably fascinating and holds promise.

## Methods

### MSCs isolation from human umbilical cord

Umbilical cords from full-term deliveries were obtained with the written informed consent of the mothers at the Obstetrics and Gynecology Unit of Cittadella Hospital and processed within 24 hours. They were collected in accordance with the requirements of the local ethical committee. The umbilical vessels were manually removed and the jelly was minced to obtain small fragments, which were plated in 100-mm petri dishes and cultured with a proliferative medium [high-glucose Dulbecco’s Modified Eagle’s Medium (DMEM) with stable L-glutamine (Euroclone S.p.a., Milan, Italy), 100 U/mL penicillin and 100 μg/mL streptomycin (Invitrogen Life Technologies, Carlsbad, USA), and 20% FBS specific for MSC cultures (Stem Cell Technologies Inc., USA)]. To allow the cells to migrate from the tissue fragments, the medium was first replaced after 5–7 days, and subsequently twice a week.

### UCMSCs characterization

The UCMSCs were analyzed by flow cytometry for the expression of the following antigens: CD14, CD29, CD34, CD45, CD71, CD73, CD90, CD105, CD166, c-kit and HLA-DR (Human Leukocyte Antigen-DR, a MHC class II cell surface receptor). As a negative control, isotype control antibodies conjugated with FITC and RPE (Santa Cruz Biotechnology, California) were used. The cytofluorimetric analysis was performed with the MoFlo High-Speed Cell Sorter (DAKO-Beckman Coulter, USA) and the data were analyzed using Summit 4.3 software (DAKO-Beckman Coulter, USA).

Mesenchymal stem cell features of UCMSCs isolated were investigated by adipogenic and osteogenic differentiation assay and their foetal origin was verified through SRY (sex-determining region *Y*) gene analysis, as previously described by our group [21].

### Human Liver Acellular Matrix (hLAM)

Surgical specimens from human livers were treated to obtain hLAM according to Meezan *et al.*[45], with minor modifications [26], on the day before each experiment.

The hLAM was analyzed by Masson trichrome staining (Bio-Optica, Milan, Italy) according to the manufacturer’s instructions and by scanning electron microscopy (SEM) to assess its structure and fibrillary components.

### *In vitro* hepatogenic differentiation on the three different supports

UCMSCs seeded on untreated plastic supports were cultured with hepatogenic differentiating medium consisting of Iscove’s Modified Dulbecco’s Medium (IMDM) (Euroclone S.p.a., Milan, Italy) supplemented with 15% FBS, 100 U/mL penicillin and 100 μg/mL streptomycin, 1% L-glutamine, 10^-7^ M dexamethasone, 10 ng/ml oncostatin M (Sigma-Aldrich, St. Louis, MO), 10 mg/ml ITS supplement (Roche Applied Sciences, Italy), 20 ng/ml hepatocyte growth factor (HGF) and 100 ng/ml fibroblast growth factor (FGF-4) (Pepro Tech EC, London, UK). Cells cultured in proliferative medium were used as a control.

The media were changed twice a week and the cells were collected at days 7, 14, 21 and 28 after induction. Immunocytochemical analyses were performed for the hepatic marker AFP and albumin. The cultures were fixed with 2% paraformaldehyde solution for 20 min at 4 °C and washed in PBS. Immunocytochemical staining was done as previously reported [20]. UCMSCs were seeded on plastic supports coated with Matrigel^TM^ (BD Biosciences Bedford, MA, USA), diluted 1:10 with DMEM, and cultured for up to 28 days, then stained as described above, or on hLAM seeded on a 24-well plate coated with hLAM and cultured as described previously up to 28 days.

### qPCR

RNA isolated from UCMSCs at each time point was reverse-transcribed and amplified with the following primers: 5′-TGACTCCAGTAAACCCTGGT-3′ and 5-AGAAATCTGCAATGACAGCC-3′ for AFP; 5′-ATCAAGAAACAAACTGCACT-3′ and 5′-GCAGGTCTCCTTATCGTCAG-3′ for albumin; 5′-CGTTCGGCATCTACTTACAGC-3′ and 5′-GTTCTCCTCCCCCTCGTCAG-3′ for MTP; 5′-GGAGAAGAAAATGAACTGCTAC-3′ and 5′-GGCTCTAAACCTGGAGT-3′ for TDO; 5′-AGCAACAGGGTGGTGGAC-3′ and 5′-GTGTGGTGGGGGACTGAG-3′ for GAPDH.

RNA isolated from mice liver was reverse-transcribed and amplified with the following primers: 5’-TCTTCTCATTCCTGCTTGTGG-3’ and 5’-GGTCTGGGCCATAGAACTGA-3’ for TNF-alpha; 5′-AAGAGAAGTGTGGCGAGGAG-3′ and 5′-CAGTTTTGTGGGGTTTTTGC-3′ for IL-5; 5’-TGGAGCAACATGTGGAACTC-3’ and 5’-GTCAGCAGCCGGTTACCA-3’ for TGF-beta 1; 5’-CCAGTTTTACCTGGTAGAAGTGATG-3’ and 5’-TTTTCACAGGGGAGAAATCG-3’ for IL-10; 5′-GATTACTGCTCTGGCTCCTA-3′ and 5′-TCGTACTCCTGCTTGCTGAT-3′ for beta-actin.

After amplification, the data were analyzed using the second-derivative algorithm. For each sample, the quantity of specific hepatic marker mRNA was expressed as n-fold the normalized amount of mRNA from undifferentiated cells.

### Albumin detection

Albumin secretion in the culture medium was quantified by ELISA according to the manufacturer’s instructions (Alpha Diagnostic Intl. Texas, USA).

### Urea assay

Urea concentration was quantified in supernatants collected from differentiated and undifferentiated cells after 28 days. 24 hours before collection of supernatants NH_4_Cl 0.3 mM was added to the medium. Urea concentration was measured by a colorimetric assay, according to the manufacturer’s instructions (Gentaur, Brussels, Belgium).

### Periodic Acid-Schiff Staining (PAS) and PAS-Diastase (PAS-D)

Cells were fixed using 4% paraformaldehyde for 10 minutes. After oxidizing in 0.5% periodic acid (Merck, NJ USA) for 5 minutes, they were treated with Schiff’s reagent (Merck, NJ USA) for 15 minutes, then rinsed in water for 10 minutes and counterstained with haematoxylin.

For PAS-D the tissue-slides were incubated with diastase solution (0.001 g/L in distilled water) in waterbath. After washing, PAS stain standard procedure was performed as described above.

### CCl_4_-induced liver injury and UCMSCs transplantation

Mice were divided randomly into five study groups with 6 animals per group. For acute CCl_4_-induced liver injury eight- week-old male Balb/c mice were injected (i.p.) with a single dose of CCl_4_ (0.75 ml/kg body weight of CCl_4_ dissolved in olive oil). The control mice received the same isovolumetric dose of olive oil as i.p. injections.

10^6^ undifferentiated UCMSCs at passage 2 resuspended in 150 μl of PBS were injected via tail vein 24 hours after CCl_4_ injection. Control animals received the same volume of PBS or UCMSCs alone. Animal care was in accordance with our institutional guidelines.

Animals were sacrificed at day 5 and day 8 after CCl_4_ injection; liver samples were collected and right lobe was fixed in 4% formalin for 24 hours and embedded in paraffin. The others lobes were stored at −80 °C until assayed.

### Histological analysis

Four-micrometer-thick liver sections were deparaffinized and rehydrated. For histological examination the slides were stained with haematoxylin and eosin. Morphometric analysis was performed measuring the percentage of necrotic areas in 6 fields versus total section area. For immunofluorescence analysis antigen retrieval was performed by microwave-heating method in 0.01 mol/L citrate buffer (pH 6). Slides were preincubated in goat serum (Sigma-Aldrich, St. Louis, MO) at 1:25 dilution in PBS for 30 minutes and then were incubated in primary antibody (monoclonal anti-alpha-SMA; monoclonal anti-human albumin, polyclonal anti-desmin and polyclonal anti-nestin, Sigma-Aldrich, St. Louis, MO) at a dilution from 1:100 to 1:2000 for 1 hour. The secondary antibody was a FITC-conjugated anti-mouse (Sigma-Aldrich, St. Louis, MO) and rhodamine-conjugated anti-rabbit (Santa Cruz Biotech., CA) at a dilution of 1:160 for 45 minutes. The section was counterstained with the nuclear marker DAPI (Vector Laboratories). Sections were imaged on Leica SP5 Confocal Microscope.

The number of positive cells was counted in 15 random fields (100x) and expressed as cells for field.

For immunohistochemistry sections were stained with anti-mouse CD68 antibody (Sigma-Aldrich, St. Louis, MO). Detection was performed after incubating the sections with horseradish peroxidase–conjugated anti-mouse antibody. Peroxidase activity was revealed by 5-minute exposure to diaminobenzidine (Sigma-Aldrich, St. Louis, MO). After washing, preparations were counterstained with Mayer’s haematoxylin for 10 minutes and mounted for analysis.

### Determination of catalase activity

Liver homogenates were prepared in phosphate buffer containing 0.5% triton (pH = 7.4). Samples were centrifuged at 12,000 x g for 30 min at 4 °C. The supernatant was collected and protein concentration was estimated by BCA^TM^ protein assay kit (Pierce Company, Rockford, IL USA).

Catalase activity was determined by measuring the exponential disappearance of H_2_O_2_ at 240 nm and expressed in units/mg of protein as described by Aebi [46].

### Statistical analyses

Data are given as median (Quartile 1; Quartile 3). The non-parametric Mann–Whitney *U*-test was used to assess differences between differentiated and control cells; time trends were evaluated using the Cuzick’s test based on Wilcoxon’s approach. Kruskal-Wallis test was used for multiple comparisons followed by the post hoc Bonferroni correction.

A p-value lower than 0.05 was assumed to indicate a significant difference (Bonferroni-adjusted at 0.016). Data analyses were performed with SPSS and StatsDirect.

## Competing interests

The authors declare that they have no competing interests.

## Authors’ contributions

PBu and FPR designed the study. DA, DB, TC, RDL, AB and ACap carried out the experimental studies. PBo collected human umbilical cord. DA, DB and SM performed the statistical analysis. PBu, DA and DB written the manuscript and MTC and PPP corrected and contributed to the final version of the manuscript. EG, ACar and UC collected human liver specimens and contributed to the interpretations of results. All authors read and approved the final manuscript.

## Pre-publication history

The pre-publication history for this paper can be accessed here:

http://www.biomedcentral.com/1471-230X/12/88/prepub

## Supplementary Material

Additional file 1 ** Figure S1.** Characterization of CCl_4_-induced liver injury. Haematoxylin and eosin stain. After 5 days from CCl_4_ administration there was a conspicuous cellular inflammatory infiltrate. After 8 days numerous cellular clusters were evident. Scale bar: 100 μm. Click here for file

Additional file 2 ** Figure S2.** Liver histology in mice control groups. Haematoxylin and eosin stain. Mice livers from control groups did not show any parenchymal abnormality. Scale bar: 100 µm. Click here for file

Additional file 3 ** Figure S3.** Stellate cells and myofibroblasts activation in CCl_4_-treated mice. Double positive alpha-SMA/desmin and alpha-SMA/nestin cells represented activated stellate cells in livers of CCl_4_-treated mice. Alpha-SMA (green) single positive cells were myofibroblasts. Scale bar: 100 μm. Click here for file
