# Supplementary figures and images for: Systemic administration of a novel human umbilical cord mesenchymal stem cells population accelerates the resolution of acute liver injury
Source: BMC Gastroenterol. 2012 Jul 12;12:88. doi: 10.1186/1471-230X-12-88 (PMC3458924; doi:10.1186/1471-230X-12-88)

**Additional file 1**

**5 days**

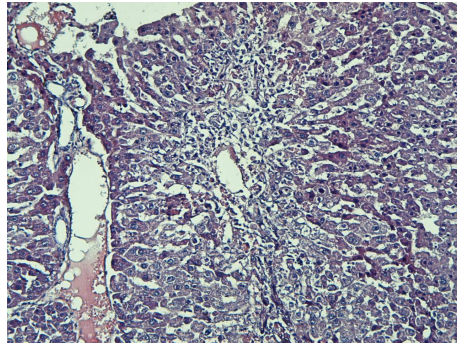

**8 days**

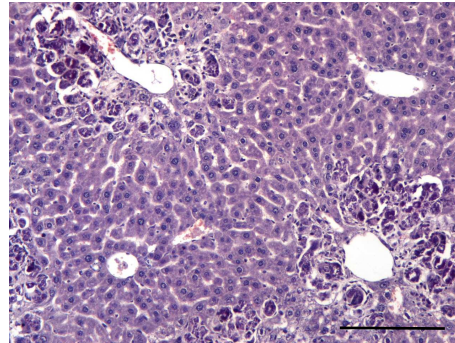

Supplement: Additional file 1 — Figure S1. Characterization of CCl4-induced liver injury. Haematoxylin and eosin stain. After 5 days from CCl4 administration there was a conspicuous cellular inflammatory infiltrate. After 8 days numerous cellular clusters were evident. Scale bar: 100 μm. [file 1471-230X-12-88-S1.pdf]

## Additional file 2

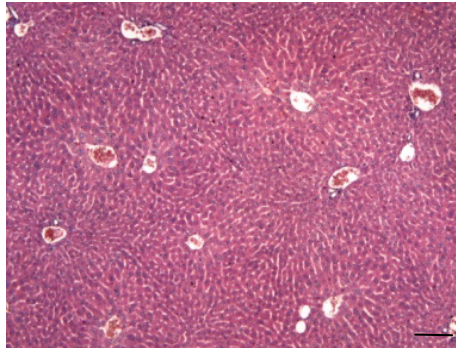

**PBS**

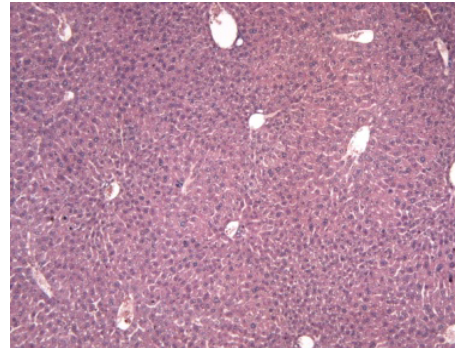

**MSCs**

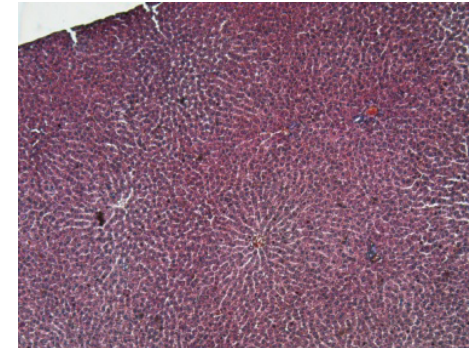

**OIL**

Supplement: Additional file 2 — Figure S2. Liver histology in mice control groups. Haematoxylin and eosin stain. Mice livers from control groups did not show any parenchymal abnormality. Scale bar: 100 µm. [file 1471-230X-12-88-S2.pdf]

### Additional file 3

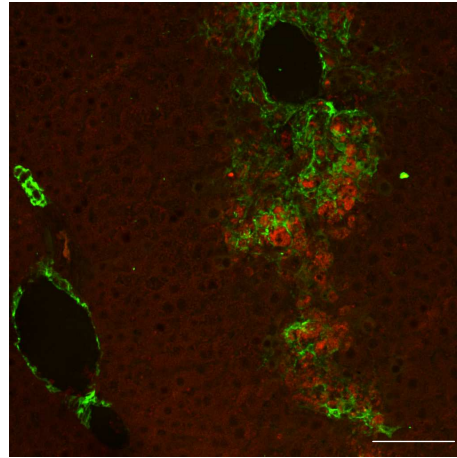

alpha-SMA/desmin

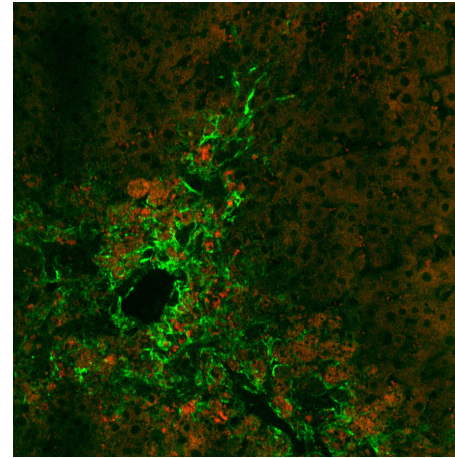

alpha-SMA/nestin

Supplement: Additional file 3 — Figure S3. Stellate cells and myofibroblasts activation in CCl4-treated mice. Double positive alpha-SMA/desmin and alpha-SMA/nestin cells represented activated stellate cells in livers of CCl4-treated mice. Alpha-SMA (green) single positive cells were myofibroblasts. Scale bar: 100 μm. [file 1471-230X-12-88-S3.pdf]
